# The impact of disease and species differences on the intestinal CLCA4 gene expression

**DOI:** 10.1007/s00109-025-02538-9

**Published:** 2025-04-12

**Authors:** K. Teske, N. A. Erickson, A. Huck, M. Dzamukova, M. Fulde, T. Heinbokel, D. Horst, N. Klymiuk, E. Pastille, A. Mekes-Adamczyk, M. Löhning, A. D. Gruber, R. Glauben, L. Mundhenk

**Affiliations:** 1 Institute of Veterinary Pathology, Faculty of Veterinary Medicine, Freie Universität Berlin, Berlin, Germany; 2 MF 3 – Animal Experimental Research and 3r – Methods Development, Research Infrastructure and Information Technology, Robert Koch Institute, Berlin, Germany; 3 Medical Department of Gastroenterology, Infectious Diseases and Rheumatology, Charité – University Medicine Berlin, Berlin, Germany; 4 Pitzer Laboratory of Osteoarthritis Research, German Rheumatism Research Center Berlin, a Leibniz Institute, Berlin, Germany; 5 Experimental Immunology and Osteoarthritis Research, Department of Rheumatology and Clinical Immunology, Charité – Universitätsmedizin Berlin, corporate member of Freie Universität Berlin and Humboldt-Universität Zu Berlin, Berlin, Germany; 6 Center of Infection Medicine, Institute of Microbiology and Epizootics, Freie Universität Berlin, Berlin, Germany; 7 Institute of Pathology, Charité – University Medicine Berlin, Berlin, Germany; 8 Large Animal Models in Cardiovascular Research, Internal Medical Department I, Technical University of Munich, Munich, Germany; 9 Center for Innovative Medical Models, Ludwig-Maximilians-University Munich, Munich, Germany; 10 Institute of Medical Microbiology, University Hospital Essen, University of Duisburg-Essen, Essen, Germany

**Keywords:** Genetic diversity, Colorectal cancer, Colitis, Inflammatory bowel disease, Epithelial-to-mesenchymal transition

## Abstract

**Abstract:**

The human chloride channel regulator, calcium-activated (CLCA) 4 is discussed as a driver of epithelial-to-mesenchymal transition as well as a biomarker for colorectal cancer (CRC) and ulcerative colitis. In contrast to humans, the *Clca4* gene is duplicated in the mouse, a common model species to study gene functions. However, the relevance of the functional murine Clca4 variants in healthy and diseased intestine is largely unknown. Here, we characterized the spatiotemporal expression patterns of the murine *Clca4a* and *Clca4b* genes in the healthy intestinal tract as well as in dextran sulfate sodium (DSS)-induced colitis and colitis-associated colon cancer (CAC) mouse model using RT-qPCR and in situ-hybridization. Similarly, we analyzed expression of the human *CLCA4* in healthy, inflamed and cancerous intestinal tracts at single cell level. Murine *Clca4a* and -*4b* but not the human *CLCA4* were detected in small intestine enterocytes of the respective species. Conversely, healthy colonocytes expressed the human *CLCA4* and its murine ortholog *Clca4a* but not the murine *Clca4b*. Under inflammatory conditions, de novo expression of *Clca4b* was observed with both murine homologs abundantly expressed in enterocytes adjacent to ulcerations. Neoplastic colonocytes expressed none or only minimal amounts of the *CLCA4* homologs both in humans and mice, whereas adjacent non-neoplastic colonocytes strongly up-regulated the human or both murine homologs, respectively. Our results suggest marked species- and homolog-specific differences in the expression patterns of the three CLCA4 homologs. Moreover, all three seem to play a role in reactive, non-neoplastic colonocytes adjacent to ulcerated and neoplastic lesions.

**Key messages:**

- Human *CLCA4* and murine *Clca4a*, but not *Clca4b*, are expressed in healthy colonocytes.
- Inflammation leads to a de novo expression of the murine *Clca4b* in colonocytes.
- Human and murine *CLCA4* homologs are absent from neoplastic enterocytes.
- Human and murine *CLCA4*s are highly expressed in tumor-adjacent, reactive colonocytes.

**Supplementary Information:**

The online version contains supplementary material available at 10.1007/s00109-025-02538-9.

## Introduction

The chloride channel regulator, calcium-activated (CLCA) 4 has been suggested to play a role in different intestinal disorders. CLCA4, at the time misidentified as CLCA2, was found to be downregulated in colorectal cancer (CRC) [1], which has been confirmed by several other studies [2–5]. Here, its low expression strongly correlated with decreased overall patient survival suggesting that CLCA4 may serve as a biomarker [3, 4, 6–8]. Experimental downregulation of CLCA4 seemed to promote epithelial-to-mesenchymal transition (EMT) of neoplastic cells, facilitating tumor growth, invasion, and metastasis [9–12]. CLCA4 has also been proposed as a biomarker for inflammatory bowel diseases (IBD) [13]. In contrast to CRC, it was up-regulated in ulcerative colitis and Crohn’s disease (CD) [13]. Nevertheless, the explicit role of CLCA4 in the healthy and diseased human intestine is largely unknown.

For research on CRC and IBD, a plethora of mouse models is available to unravel mechanisms of inflammatory and neoplastic conditions of the intestine [14–16]. In general, species-specific differences between humans and mice should be respected and experimental results only carefully translated to humans. Ideally, such translations rely on a thorough understanding of these differences, many of which, however, are still unknown.

In this regard, the *CLCA* gene family is particularly diverse among vertebrates. Varying numbers of genes, putative functions and cellular expression patterns have been found between different mammalian species [17, 18]. In particular, most notable differences have been revealed between human and murine *CLCA4* homologs [17]. While the human genome contains only a single *CLCA4* gene, the mouse possesses three distinct *Clca4* homologs [17]. Two of these, *Clca4a* and -*4b*, formerly known as *mClca6* and *− 7*, respectively, are putatively functional, whereas *Clca4c*, alias *mClca8*, seems to represent a pseudogene, as predicted by its premature stop codons [19]. Human *CLCA4* mRNA has been detected in several tissues [10, 11, 20–22]. The expressing cell type has so far only been revealed for the intestine, localized to epithelial cells of the colon [12, 22]. Murine *Clca4a* and *− 4b* were both found in the intestinal tract [23, 24], in which enterocytes [25] of the small and large intestine express *Clca4a* whilst the cell types expressing *Clca4b* are unknown.

The cellular expression patterns of the murine Clca4 members in healthy and diseased intestine and, in particular, their roles in relation to the single human CLCA4 are unknown to date. However, this information may be crucial when using mouse models to further investigate the role of CLCA4 as, for example, a modulator of EMT or biomarker in inflammatory disorders.

Therefore, the aim of this study was to characterize the tissue and cellular expression patterns of the two putatively functional murine *Clca4* homologs in healthy, inflamed, and tumorous intestines. Their cellular expression patterns were determined in healthy intestinal segments at various developmental stages. We extended our analyses to intestines of murine models of CRC and IBD. Finally, human *CLCA4* was localized in the cellular microenvironment of CD and CRC compared to segments of the healthy intestine. Systematic comparisons between the three homologs revealed both similarities and major differences between the species and between healthy *versus* diseased environments.

## Materials and methods

### Ethics statement, animals, and sample collection

Euthanasia of mice was in accordance with the FELASA guidelines and acknowledged by the local governmental authorities (State Office of Health and Social Affairs Berlin, IDs: T 0181/15, T 0104/06). Adult female and male untreated C57BL/6 J mice 6 to 54 weeks of age had been sacrificed for other scientific purposes (ID: T 0104/06) and archived tissues of P10 to P30 (10-, 20- and 30-day-old) mice (ID: T 0181/15) were used in accordance with the 3R principle. Tissue samples of the gastrointestinal tract were snap frozen in liquid nitrogen and stored at − 80 °C. Samples for Laser Capture Microdissection were stored in Tissue-Tek O.C.T. Compound Medium (Sakura, USA) at − 80 °C. The same set of samples was also fixed in 4% formaldehyde solution and embedded in paraffin. The following segments of the gastrointestinal tract were sampled in adult mice: nonglandular and glandular stomach, duodenum, proximal and distal jejunum, ileum, caecal corpus and apex, proximal, middle, and distal colon, and rectum. In P10, P20, and P30 mice, nonglandular and glandular stomach, duodenum, jejunum, ileum, caecum, colon, and rectum were sampled.

For the analysis of inflamed tissues, formalin-fixed, paraffin-embedded (FFPE) as well as cryopreserved colon tissues of a previously approved and published murine dextran sodium sulfate (DSS) colitis study (State Office of Health and Social Affairs Berlin, approval ID: G 0170/12) [26] were re-used (n = 10, controls n = 3; C57BL/6 J) in accordance with the 3R principle. For the analysis of tumorous tissues, cryopreserved tissue samples of distal neoplastic and proximal non-neoplastic colon tissues as well as FFPE samples of distal neoplastic colon tissues of a previously approved and published murine CAC model (State Agency for Nature, Environment and Consumer Protection in North Rhine-Westphalia, approval ID: 81–02.04.2017.A445) [27] were also re-used (CAC model n = 5 of FFPE samples, n = 6 of cryopreserved samples, healthy controls n = 3; BALB/c). Neoplastic areas at the center of the tumor and non-neoplastic regions at the apical surface of the tumor, at the border between tumor and non-neoplastic area, and tumor-adjacent non-neoplastic tissues were evaluated for cellular expression of *Clca4* homologs.

Additionally, samples of histologically unaffected small and large intestine (n = 3, FFPE) from humans 56 to 83 years of age as well as samples of CRC (n = 3) from humans 58 to 90 years of age were included (approved by the ethics committee of Charité – University Medicine Berlin, approval ID: EA1/039/24) and analyzed analogously to the murine samples.

### Quantitative RT-PCR

Extraction of total RNA, reverse transcription and quantitative polymerase chain reaction (RT-qPCR) were conducted as described [28]. Transcription levels of *Clca4a* and -*4b* were normalized to the reference genes *eukaryotic translation elongation factor- 1α* (*Ef- 1α*), *b- 2 microglobulin* (*B2 m*), and *glyceraldehyde- 3-phosphate dehydrogenase* (*Gapdh*). *Hypoxanthine phosphoribosyltransferase 1* (*HPRT*) was used as reference gene in postnatal expressional analyses. ΔCt was determined as ΔCt = Ct (gene of interest)—Ct (mean of reference genes). Primers and probes are listed in Tab. S1.

### In Situ*-*Hybridization (ISH)

ISH was performed as reported [29] using the ViewRNA ISH Tissue Assay Kit (Invitrogen by Thermo Fisher Scientific, Darmstadt, Germany) following the manufacturer’s instructions with minor adjustments. Probes for the detection of murine *Clca4a*, -*4b*, the human *CLCA4*, and the respective species-specific *EF- 1α* reference genes were designed by the manufacturer. 4 µm thick FFPE tissue sections were mounted on adhesive glass slides, dewaxed in xylene, and rehydrated in ethanol. Tissues were incubated at 95 °C for 10 min with subsequent protease digestion for 20 min. Sections were fixed with 4% paraformaldehyde in phosphate-buffered saline (Alfa Aesar, Thermo Fisher, Kandel, Germany) and hybridized with the respective probes. Amplifier and label probe hybridizations were performed using fast red as chromogen, followed by counterstaining with hematoxylin for 45 s, washing in tap water for 5 min, and mounting with Roti-Mount Fluor-Care DAPI (4, 6-diaminidino- 2-phenylindole; Carl Roth). Negative and morphological controls were obtained by omitting probes or pretreatment, respectively. Slides with positive signals for the reference genes were analyzed and images obtained using an Olympus BX41 microscope with a DP80 Microscope Digital Camera and the cellSens Imaging Software, Version 1.18 (Olympus Corporation, Münster, Germany).

### Laser capture microdissection

Tissue samples stored in Tissue-Tek O.C.T. Compound Medium were cryosectioned at − 16 °C in a cryostat chamber and − 18 °C knife temperature. Sections of 10 µm thickness were transferred to 2 μm polyethylene naphthalate-membrane glass slides (Leica Microsystems, Germany). Laser microdissection was performed using Leica LMD7 device and samples were captured in 30 μl lysis buffer (ARCTURUS PicoPure RNA Isolation Kit). An area of approximately 1,000,000 µm^2^ was collected of each of the following intestinal locations: jejunal villi, jejunal crypts, colon apical and crypt area. Subsequently, the samples were briefly centrifuged, incubated at 42 °C for 15 min followed by brief vortexing and snap freezing on dry ice for storage at − 80 °C until further processing. Total RNA was isolated using ARCTURUS PicoPure RNA Isolation Kit according to manufacturer's instructions. 9 μl of each sample were reverse transcribed in 20 μl reaction volume using Iscript (BioRad) according to manufacturer’s instructions. RT-qPCR was performed as described above.

### Statistics

Following control of normal distribution of the data obtained from healthy and tumorous colon tissues of the CAC model by the Shapiro–Wilk-Test, data were compared using the unpaired two-tailed t-test with a 95% confidence interval. Data from the DSS colitis model were statistically analyzed by the Mann–Whitney *U* test. P < 0.05 was considered significant. All statistical analyses and graphical illustrations were performed using GraphPad PRISM 6 (GraphPad Software Inc., La Jolla, USA). RT-qPCR data are displayed as single value fold change with fold changes of 0.5 and 2 set as limits for valid statement of lowered or elevated expressions, respectively.

Analysis of published single cell RNA-sequencing (sc-RNA seq) data.

CD data [30] were downloaded from the Broad Single Cell Portal (accession number SCP1884). Data of CRC patients [31] were accessed from Zenodo [32]. Provided normalized expression values were further processed using the Seurat-package (v 5.0.3) in R (v. 4.2.0). Regarding the CD dataset, original clusters were merged into major cell populations (e.g., enterocytes or stem cells) while for the CRC data, original cell clusters were used. Mean CLCA4 expression per cell population and frequency of CLCA4^+^ cells per cluster were calculated using Seurat’s dotplot-function and visualized using ggplot2 (v. 3.5.0).

## Results

### Expression of *Clca4a* and − *4b* in enterocytes of the small and large intestine

**Fig. 1:**
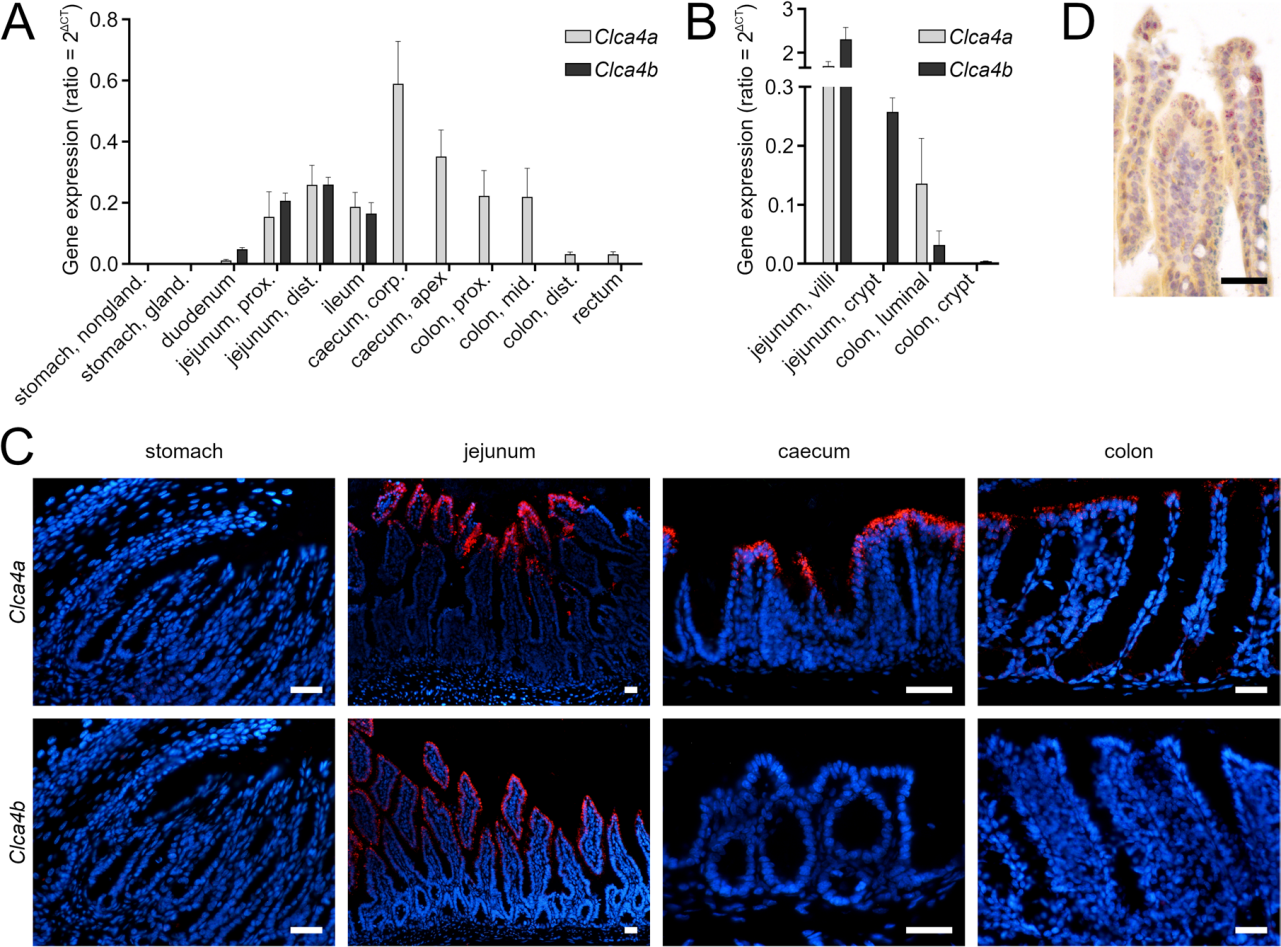
**A** In adult mice, both *Clca4a* and -*4b* are expressed on mRNA level in the small intestine, whereas only *Clca4a* is expressed in the large intestine. Relative expression levels of *Clca4a* and -*4b* were determined by RT-qPCR with *Ef- 1α*, *ß2M*, and *GAPDH* as reference genes. Neither *Clca4a* nor -*4b* were expressed in the stomach. In the small intestine, *Clca4a* and -*4b* were expressed to a comparable degree. In the large intestine, *Clca4b* was not expressed whereas *Clca4a* was highly expressed, particularly in the caecum. Data are expressed as mean ± SEM and expression levels are provided in ratios of *Clca* to the reference genes *Ef- 1α*, *ß2M*, and *GAPDH*. Ct = cycle threshold, prox. = proximal, mid. = middle, dist. = distal. Adult mice; n = 4. **B** The expression of *Clca4a* and -*4b* varies along the crypt-villus axis. The mRNA expression of *Clca4a* and -*4b* was quantified by RT-qPCR in laser capture microdissected samples from specific intestinal locations. Both homologs were found expressed in the jejunal villi, whereas only *Clca4b* was also expressed in the jejunal crypts. In the colon, *Clca4a* was exclusively detected in the luminal areas but not in the crypts. Using this technique, traces of *Clca4b* transcripts were detected in both colon locations investigated. Data are expressed as mean ± SEM and expression levels are given in ratios of *Clca* to reference the genes *Ef- 1α*, *ß2M*, and *GAPDH*. Ct, cycle threshold. Adult mice; n = 3. **C ***Clca4a* and -*4b* were expressed in intestinal epithelial cells. mRNA of both *Clca4a* (red, top panel) and -*4b* (red, bottom panel) was detected in enterocytes of the jejunum via ISH. In contrast, only *Clca4a* but not *Clca4b* was detected in enterocytes of the large intestine. The stomach was devoid of *Clca4a* or -*4b* expression. Adult mice; n = 3. Blue = Roti®-Mount Fluor-Care DAPI nucleus staining. Scale bars = 50 μm. **D** *Clca4a* and -*4b* showed a regionally shifted expression pattern in enterocytes of the jejunal villi. Along the crypt-villus axis, *Clca4a* (red) and -*4b* (blue) were both expressed by the same enterocytes, however, only in the central and the apical areas of the villi. In contrast, enterocytes of the villi below these areas exclusively expressed *Clca4b* (blue). Duplex ISH; adult mouse. Scale bar = 50 μm

Upon quantification of mRNA expression in whole tissue lysates of the gastrointestinal tract, neither *Clca4a* nor *− 4b* were detected in the stomach (Fig. 1A). In the small intestine, *Clca4a* and *− 4b* were expressed at similar levels (Fig. 1A) whereas in the large intestine, only *Clca4a* was expressed with the highest relative expression levels located in the caecum. Along the crypt-villus axis, both genes were detected at similar levels in the jejunal villi, however, the crypts exclusively expressed *Clca4b* (Fig. 1B). In the colon, *Clca4a* was detected in the apical half of the mucosa but not in the crypts. *Clca4b* was expressed only marginally in the apical mucosa. At single cell resolution, strong mRNA signals of both genes were obtained in enterocytes at the tips of virtually all jejunal villi (Fig. 1C). *Clca4a* but not *Clca4b* was distinctively expressed in virtually all enterocytes at the apical mucosal lining of the caecum and colon but not in the crypts. In order to specify whether *Clca4a* and *− 4b* are expressed in the same cells of the jejunal villus tips, both mRNA sequences were colocalized via ISH showing widely overlapping cellular expression of both homologs in numerous apical enterocytes (Fig. 1D). However, exclusively *Clca4b* mRNA was also detected in enterocytes closer to the basal areas of the villi.

### Distinct expression of *Clca4a* and − *4b* during early postnatal development

**Fig. 2:**
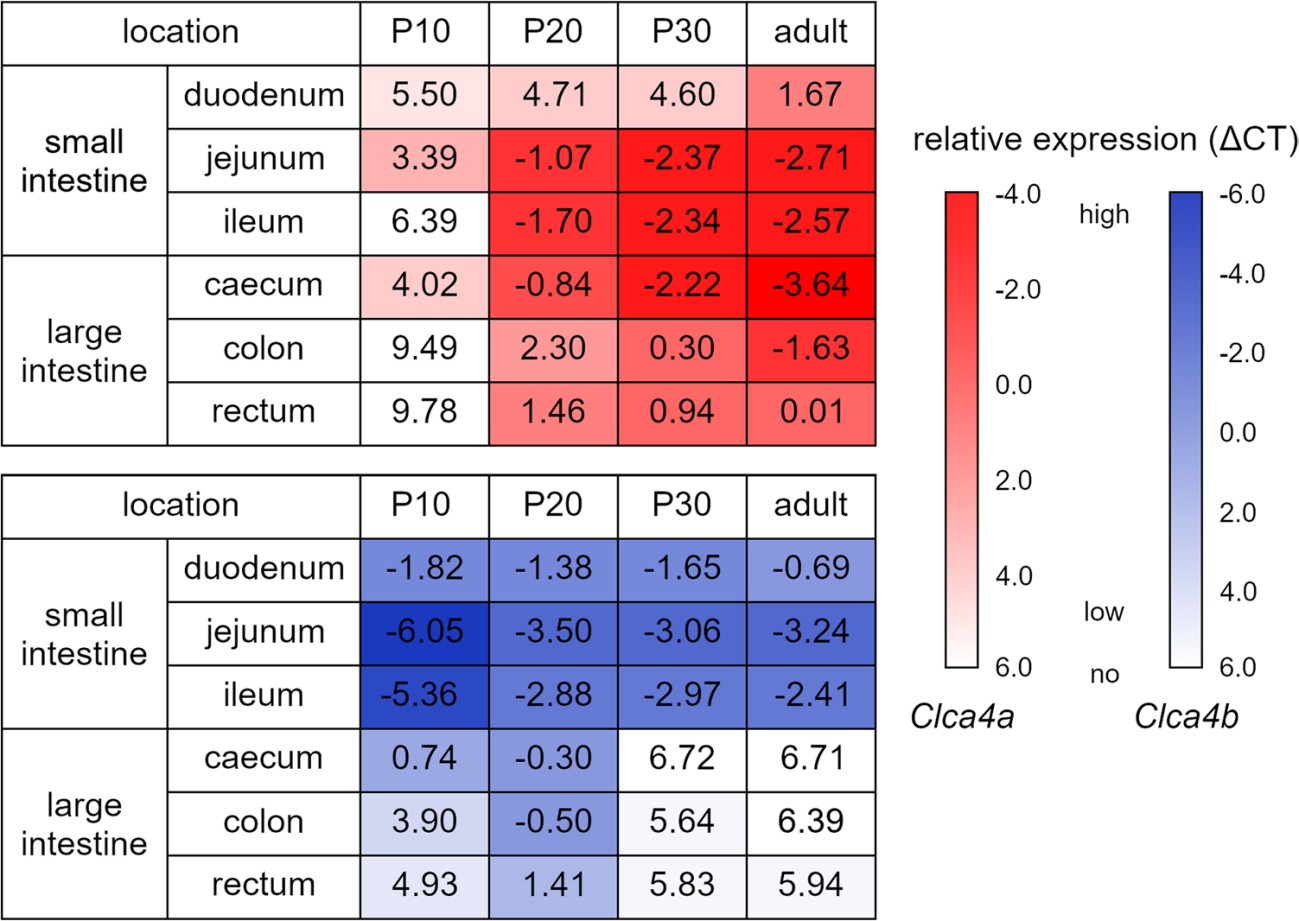
Murine *Clca4a* and -*4b* showed an age-dependent expression profile in the gastrointestinal tract. Relative expression levels of *Clca4a* (red color shading, top) in the small and large intestine increased with age, as determined by RT-qPCR. In contrast, *Clca4b* (blue color shading, bottom) showed similar relative expression levels of all ages analyzed in the small intestine. In the large intestine, its expression increased until P20 and was undetectable thereafter. *HPRT* was used as reference gene. Ct, cycle threshold. ΔCt = Ct (gene of interest)—Ct (reference gene). Ct values of 6 and higher were interpreted as no expression. n = 4 to 7

In P10 mice, *Clca4a* was detected at low levels in select intestinal segments (Fig. 2). Its presence both in the small and large intestine increased with age, rendering highest expression levels in adult mice. In contrast, *Clca4b* was already strongly expressed in the small intestine of P10 mice, slightly decreased in P20 and remained rather constant at later ages. In the large intestine, its expression increased from P10 to P20 and became undetectable at P30 and thereafter.

### De novo expression of murine *Clca4b* in colonocytes of a colitis model

**Fig. 3:**
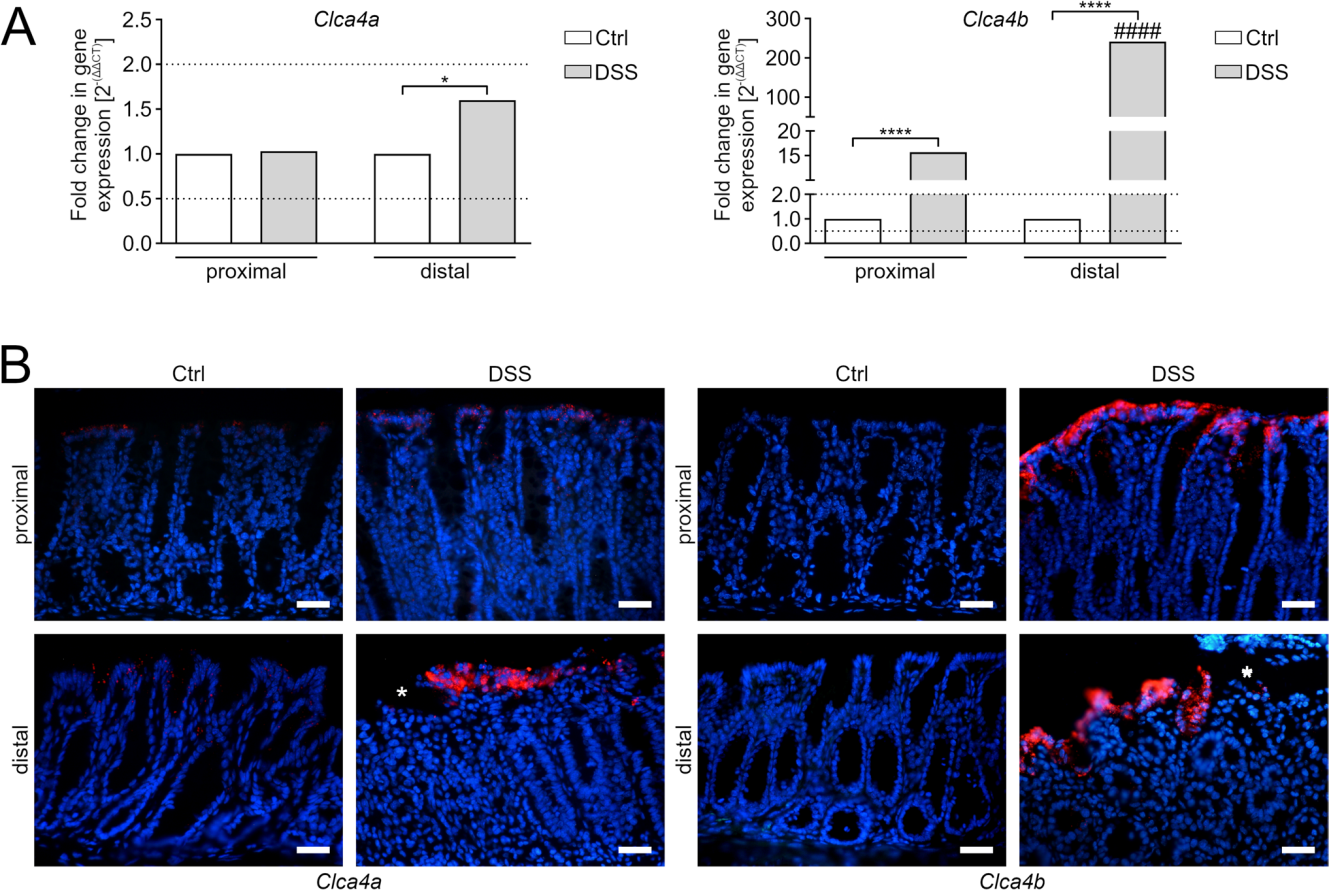
**A** Abundant de novo expression of *Clca4b* in colon tissue of a murine DSS colitis model. The expression of *Clca4a* and -*4b* was quantified in proximal and distal colon of a murine DSS colitis model by RT-qPCR. Left – *Clca4a* expression did not show any difference between DSS colitis and healthy controls in the proximal colon. However, *Clca4a* was significantly upregulated in the distal colon under conditions of DSS colitis. Right – *Clca4b* expression significantly increased in proximal and also in distal colon in DSS colitis. Data are expressed as mean fold change and *Ef- 1α*, *ß2M*, and *GAPDH* were used as reference genes. Ct, cycle threshold. Dotted lines indicate fold changes of 0.5 and 2 as limits for valid statement of lowered or elevated expression, respectively. n = 10 per group. *p < 0.05 and ****p < 0.0001 vs. Ctrl and ^####^*p* < 0.0001 vs. proximal colon by Mann–Whitney-U test. **B** *Clca4a* and -*4b* were markedly expressed in enterocytes of DSS-challenged mice. *Clca4a* (red, left panel) was expressed in the distal colon of DSS-challenged mice compared to healthy controls as determined via ISH. *Clca4b* (red, right panel) was also strongly expressed in both proximal and distal colon whilst being absent from healthy controls. The most prominent signals were detected in enterocytes adjacent to acute ulcerations (*). Murine DSS colitis model; n = 3. Blue = Roti®-Mount Fluor-Care DAPI nucleus staining. Scale bars = 20 μm

Expressions of *Clca4a* and -*4b* were subsequently analyzed in a DSS colitis model. In whole tissue lysates, *Clca4a* was only slightly upregulated in the distal colon of DSS colitis mice compared to healthy controls (Fig. 3A). In the proximal colon, however, no differences were observed. In contrast, *Clca4b* was massively upregulated in both proximal and distal inflamed murine colon (Fig. 3A). The cellular expression pattern as detected by ISH mirrored these results. *Clca4a* was strongly expressed in most apical enterocytes of the distal colon but weakly in the proximal colon during DSS colitis with low expression in both regions in unchallenged mice (Fig. 3B). In contrast, *Clca4b* showed a strong de novo expression in the proximal and distal colon under inflamed conditions but no expression in unchallenged mice (Fig. 3B). Of note, intense staining for both *Clca4a* and -*4b* was observed adjacent to ulcerated mucosal areas (Fig. 3B, bottom right images of both panels).

### Distinctively strong expression of *Clca4a* and − *4b* adjacent to colon cancer cells

**Fig. 4:**
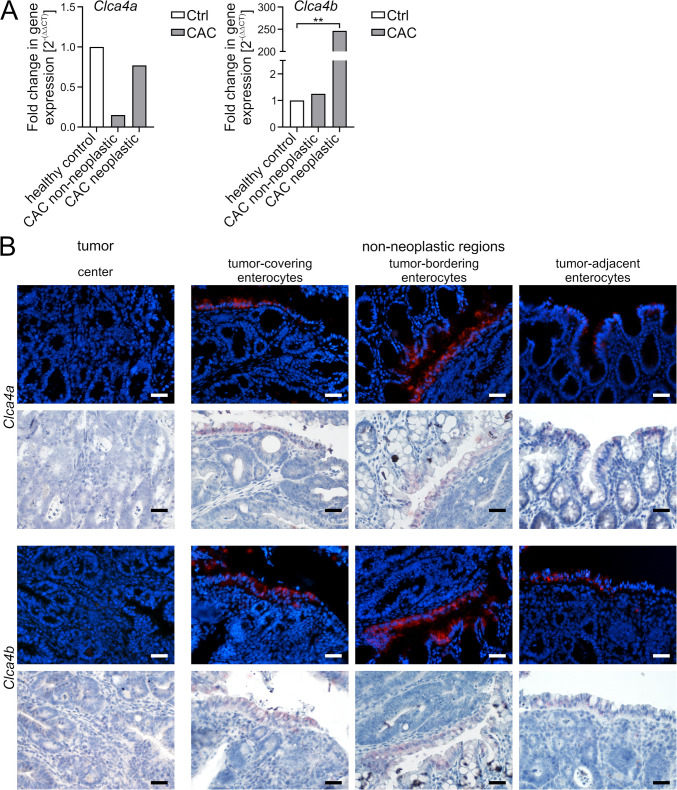
**A** De novo expression of *Clca4b* in whole tissue lysates of a colitis-associated colon cancer (CAC) model. *Clca4a* expression levels (left panel) failed to show any statistically significant differences between neoplastic or non-neoplastic intestinal regions of the CAC model and healthy controls, although faintly downregulated in non-neoplastic CAC colon samples, as determined by RT-qPCR. *Clca4b* expression (right panel) was significantly increased in the neoplastic CAC colon. Data are expressed as mean fold change and *Ef- 1α*, *ß2M*, and *GAPDH* were used as reference genes. Ct, cycle threshold. n = 3 to 6 per group. **p < 0.01 vs. Ctrl by unpaired two-tailed t-test. **B** In the colitis-associated colon cancer (CAC) model, *Clca4a* and -*4b* were predominantely expressed in enterocytes at the junction between tumor and non-neoplastic area, but not in the tumor itself. In most animals, no expression of *Clca4a* or -*4b* was detected via fluorescence ISH in the center of the neoplastic tissue. *Clca4a* (red, top panel) was moderately to strongly expressed at the tumor surface and border and only minimally to moderately in the tumor-adjacent non-neoplastic area. *Clca4b* (red, bottom panel) was not expressed at the tumor surface, however, it showed a moderate to strong expression at the border and a moderate expression in the non-neoplastic adjacent area. Murine CAC model, n = 5. Blue = Roti®-Mount Fluor-Care DAPI nucleus staining. Scale bars = 20 μm. Lower panels show the corresponding bright field images of the sections, red signals of chromogenic ISH = *Clca4a* and -*4b*, respectively

*Clca4a* and -*4b* expression was also analyzed in a CAC model. No differences in *Clca4a* expression were detected between healthy and neoplastic tissues using quantitative whole tissue lysate analysis (Fig. 4A). *Clca4a* seemed slightly downregulated in the non-neoplastic CAC samples, however, without statistical significance (Fig. 4A). In contrast, *Clca4b* was massively upregulated in the neoplastic regions, whereas no regulatory differences were detected in non-neoplastic regions of the CAC tissue and healthy controls (Fig. 4A).

Furthermore, the cellular expression patterns of *Clca4a* and -*4b* were characterized in distinct CAC tissue areas using ISH (defined in Fig S2). No expression of *Clca4a* was detected in the tumor masses (Fig. 4B, Tab. S3A). Similar results were observed for *Clca4b* in four of five mice (Fig. 4B, Tab. S3B). Only a single mouse had weak *Clca4b* signals in few tumor cells (Fig. S4, Tab. S3B). Of note, prominent signals of *Clca4a* and -*4b* were found in apical non-neoplastic enterocytes of the tumor surface, directly at the transition to non-neoplastic areas and in close proximity to the tumor in all samples investigated (Fig. 4B, Tab. S3). In general, *Clca4b* seemed more strongly expressed than *Clca4a* in these regions.

### Expression of human *CLCA4* in enterocytes of the large intestine.

**Fig. 5:**
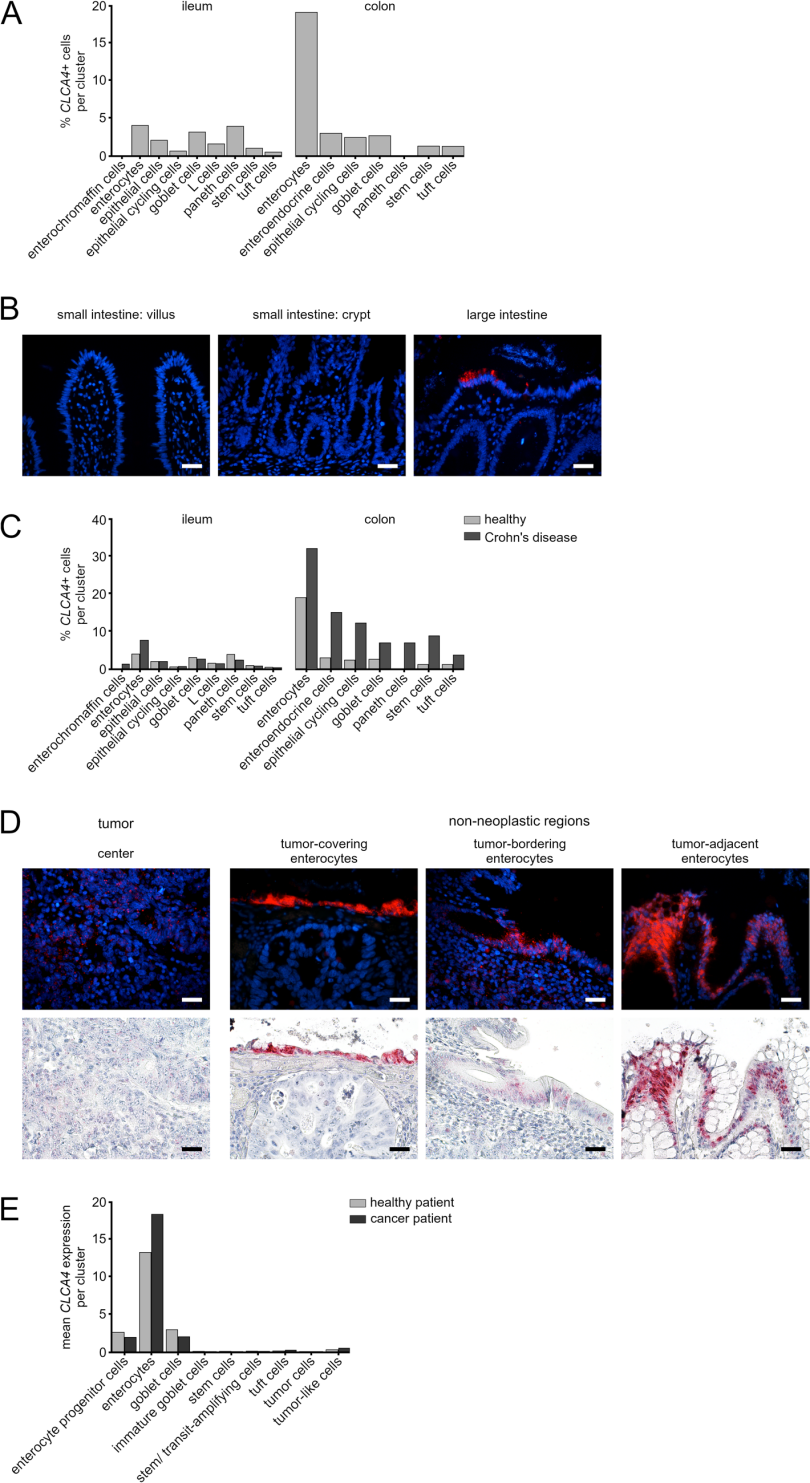
**A** Human *CLCA4* mRNA was predominantly detected in colonic enterocytes. Previously published [30] sc-RNA seq data were used to calculate the percentage of CLCA4^+^ cells per cell population. **B** Human CLCA4 mRNA was expressed in enterocytes of the large but not small intestine. ISH revealed a moderate, patchy *CLCA4* expression (red) in luminal enterocytes of the large intestinal epithelium, whereas the small intestine lacked *CLCA4* expression. Adult humans; n = 3. Blue = Roti®-Mount Fluor-Care DAPI nucleus staining. Scale bars = 20 μm. **C** In CD, an increase in frequency of CLCA4 + cells was found for all cell populations in the colon but only for enterocytes in the ileum. The same dataset as in **A** was used to calculate the percentage of CLCA4 + cells in healthy individuals and CD patients. **D** Cellular expression pattern of *CLCA4* in colorectal cancer. Only scattered *CLCA4* signals were found in the tumor cells by fluorescence ISH. In contrast, moderate to strong patchy signals were observed in the non-neoplastic epithelium adjacent to the tumor and, in particular, at the junction between tumor and healthy tissue. Adult humans; n = 3. Blue = Roti.®-Mount Fluor-Care DAPI nucleus staining. Scale bars = 20 μm. Lower panel shows the corresponding bright field images of the sections, red signals of chromogenic ISH = *CLCA4*. **E** In human CRC, increased expression of *CLCA4* was found in non-malignant enterocytes from tumorous tissue samples compared to enterocytes from adjacent healthy tissue. Tumor cells failed to show *CLCA4* expression. Mean *CLCA4* expression was calculated per cell cluster; data were taken from a previously published sc-RNA seq dataset of human CRC samples [31]

Previous studies had investigated the cellular expression pattern of human *CLCA4* only in the colon but not the small intestine [12, 22]. Analysis of previously published data [30] from sc-RNA seq revealed four-fold frequency increase of CLCA4^+^ colonic enterocytes compared to ileal enterocytes or other cell types (Fig. 5A). This was underlined by ISH results, where *CLCA4* expression had a patchy pattern in apical enterocytes of the large but not the small intestine in histologically unaffected samples (Fig. 5B).

### Increase of CLCA4^+^ cells in the colon of CD patients

In CD patients, increased numbers of CLCA4^+^ cells were detected among the enterocytes of the colon and ileum and other cell types of the colon compared to healthy controls (Fig. 5C). While a distinct increase in CLCA4-expressing cells was observed in all large intestinal cell populations analyzed, this increase was limited to enterocytes in the ileum.

### Similar expression of human *CLCA4* in colorectal cancer and its orthologs in a murine cancer model

In CRC, minimal expression of *CLCA4* was detected at single cell resolution in scattered tumor cells of two out of three patients analyzed (Fig. 5D, Tab. S5). In contrast, and similar to mice, a moderate to strong patchy expression of *CLCA4* was detected in all samples of apparently non-neoplastic colonocytes apical to the tumor and especially at the transition zone between tumor and adjacent healthy tissue as well as in close proximity to the tumor. These data are in concordance with previously published sc-RNA seq data [31], which had shown a strong expression of *CLCA4* for non-neoplastic enterocytes in tumorous regions, i.e. of healthy signature, compared to enterocytes from adjacent healthy mucosa in non-tumorous regions (Fig. 5E). However, no *CLCA4* expression had been detected in tumor cells, stem cells, or immature cells. This activation of expression appears to relate to increased expression per cell and is not merely the result of de novo expression of *CLCA4* in these cell populations (Fig. S6).

## Discussion

A thorough understanding of species-specific differences is of key importance when employing mouse models in functional genomics or other comparative investigations. The human *CLCA4* gene product has been proposed as modulator of EMT and biomarker in colitis and CRC [2–4, 6–8, 11–13]. However, a comprehensive intestinal expressional analysis of its murine *Clca4* homologs has been lacking. Here, our comparative interspecies analysis revealed differences but also similarities, particularly under conditions of disease.

In enterocytes of the healthy small intestine, both murine *Clca4* members were detected at similar expression levels, whereas in humans, *CLCA4* was not expressed. We can only speculate that factors such as differences in diet, microbiomes, or immunological factors necessitate the molecule´s presence in the small intestine of mice only. In any case, more influential factors or conditions may not have been represented by the small human sample number analyzed here.

In contrast to the small intestine, two striking features of *CLCA4* variant expression in the large intestine were found. First, *CLCA4* was detected in colonocytes of both species. Of note, all murine colonocytes seem to uniformly express *Clca4a*, whereas human *CLCA4* expression appeared patchy. While the factors behind this difference and its putative functional significance remain unclear, CLCA4 may be indispensable in the colon in both species.

Second, *Clca4a* but not -*4b* was expressed in the healthy murine large intestine, suggesting that adult mice require both *Clca4* homologs in the small but only *Clca4a* in the large intestine. Sub- or even neofunctionalization is a known sequela of gene duplication events [33, 34]. Such a scenario may also apply to the duplication of murine *Clca4* with subsequent functional diversification of Clca4a and − 4b in their respective cellular environment. The differences in postnatal expression and along the jejunal crypt-villus axis of both homologs may also point towards functional heterogeneity. Therefore, comparative functional studies of the homologs and also orthologs are warranted which may reveal transcriptional, functional or regulatory differences.

Under conditions of colitis, both the expression levels and cellular expression patterns of murine *Clca4a* and -*4b* were clearly altered. In the DSS colitis model, *Clca4a* was more strongly expressed in enterocytes of challenged than of healthy mice, in particular at the transition zone between inflamed to ulcerated areas (Fig. 3). Furthermore, *Clca4b* showed a massive de novo expression in enterocytes of the inflamed large intestine. Hence, both murine *Clca4* members seem to be induced by inflammatory conditions similar to the scenario in CD (Fig. 5C) and the reported upregulation of its human ortholog in IBD patients [13]. Particularly *Clca4b* may be useful as a biomarker for colitis as had been postulated for its human ortholog [13]. Interestingly, inflammation seems to trigger the expression and, hence, both murine Clca4 variants appear to play an as yet undefined role in the inflamed colon.

Under tumorous conditions, no or only marginal expressions of *Clca4a* and -*4b* were detected in tumor cells of a murine model of CRC (Fig. 4) as well as *CLCA4* in the human CRC (Fig. 5D, E). This is in line with several previous studies on CRC in which human CLCA4 had been had been found down-regulated in tumor tissues [2–5]. In contrast to tumor cells, murine *Clca4a* and -*4b* as well as their human ortholog were highly expressed in adjacent non-tumorous enterocytes, which likely explains the apparent up-regulation of *Clca4b* in whole tissue lysates of tumor samples, likely including its margins (Fig. 4A).

Our observation that inflammation and also neoplastic conditions apparently trigger the expression of the *CLCA4* homologs in murine and human enterocytes may be useful to elucidate the driving mechanism of this induction and the pathophysiological consequence of its overexpression in the cellular microenvironment. Tissue regeneration may be an explanation. To date, the basic function of *CLCA4* remains speculative but our data support previous hypotheses on its role in epithelial differentiation [9–12]. Specifically, gain-of-function cell models had shown that the primary mesenchymal morphology was altered in favor of a more pronounced epithelial morphology and reduced invasion and migratory capacity of these cells [11, 12]. The observed expressional induction of the *Clca4* homologs in enterocytes in diseased tissues and the lack of expression in tumor cells would be in line with the supposed role in epithelial differentiation. The expression pattern along the crypt-villus axis may also point towards its putative function, considering that the adult small and large intestinal crypts represent the proliferative compartment consisting of undifferentiated cells, whereas the apical compartment contains differentiated cells [35]. However, several other functions of the CLCA4 homologs are conceivable which warrant further comparative functional studies.

Taken together, this study clearly highlights that the genetic diversity of *CLCA4* genes in humans and mice is accompanied by some interspecies similarities but also clear differences in intestinal expression patterns. The duplication of *Clca4* in mice and their homolog-specific expressions in the healthy intestine and under conditions of disease render the mouse a complex model to study the role of CLCA4 in human intestinal physiology and pathology. Regardless of their functions yet to be elucidated, such species-specific differences should be considered when mouse models are employed to study human intestinal diseases.

## Supplementary Information

Below is the link to the electronic supplementary material.

Supplementary file1 (DOCX 25 KB)

Fig. 6 S2. Figure – Depicted locations of cellular expression analysis of CLCA4 homologs in the CAC model. Yellow = tumor (center), green = non-neoplastic tumor-covering enterocytes, purple = non-neoplastic tumor-bordering enterocytes, red = non-neoplastic tumor-adjacent enterocytes. The same regions were analyzed in human CRC samples. Lower image = unannotated image, hematoxylin stained. Scale bars = 50 μm

High resolution image (TIF 9489 KB)

Supplementary file3 (DOCX 16 KB)

Fig. 7 S4.Figure – While no expression of *Clca4b* was detected in the depths of the neoplastic tissue in four mice with CAC via ISH, only a single mouseshowed moderate, diffuse *Clca4b* signals (red) in the tumor. Murine CAC model. Blue = Roti^®^-Mount Fluor-Care DAPI nucleus staining. Scale bar = 20 μm.

High resolution image (TIF 4242 KB)

Supplementary file5 (DOCX 14 KB)

Fig. 8 S6. Figure - Percent of CLCA4 positive cells per cell population in human CRC compared to heathy adjacent tissue. Previously published [30] sc-RNA seq data were used for the calculation

High resolution image (TIF 8816 KB)

## Data Availability

All relevant data are within the paper and its Supporting Information files.
