# Supplementary material for: The impact of disease and species differences on the intestinal CLCA4 gene expression
Source: J Mol Med (Berl). 2025 Apr 12;103(6):687–97. doi: 10.1007/s00109-025-02538-9 (PMC12141163; doi:10.1007/s00109-025-02538-9)
Supplement: Supplementary file 1 — Supplementary file1 (DOCX 25 KB) [file 109_2025_2538_MOESM1_ESM.docx]

SUPPORTING INFORMATON

Table S1

Quantitative Real Time RT-PCR and conventional PCR: Sequences and Specifications

| Gene | Genbank  Accession no. | Oligonucleotide Sequences (5’-3’) | Amplicon  Size, bp | Annealing Temp., °C |
| --- | --- | --- | --- | --- |
| *mClca4a* [1] | NM_207208 | Primer (upstream) CATAATAAAAAGTGCAATTACAG  Primer (downstream)CTTGGGAAGTAATCAGCACTTCTG  TaqMan Probe FAM-AGGGTGACGTCTCCAT-BHQ | 91 | 60 |
| *mClca4b* [1] | NM_001033199.3 | Primer (upstream) ATGGAGACACCACCGG  Primer (downstream)CTTCTTCTCCCTGCTGAGGATCAG  TaqMan Probe FAM-TCTATTCAGACGGTCAGAAGAAC-BHQ | 114 | 60 |
| *Ef1a* [2] | NM_010106.2 | Primer (upstream) AAAAACGACCCACCAATGG  Primer (downstream)GGCCTGGATGGTTCAGGATA  TaqMan Probe FAM-AGCAGCTGGCTTCACTGCTCAGGTG-BHQ | 67 | 60 |
| *B2m* [3] | NM_009735.3 | Primer (upstream) ATTCACCCCCACTGAGACTGA  Primer (downstream)CTCGATCCCAGTAGACGGTC  TaqMan Probe FAM-TGCAGAGTTAAGCATGCCAGTATGGCCG-BHQ | 86 | 60 |
| *Gapdh* [4] | NM_008084.2 | Primer (upstream) TCACCACCATGGAGAAGG  Primer (downstream)GCTAAGCAGTTGGTGGTGCA  TaqMan Probe FAM-ATGCCCCCATGTTTGTGATGGGTGT-BHQ | 169 | 60 |
| *HPRT* [5] | NM_013556.2 | Primer (upstream) GGTTAAGCAGTACAGCCCCA  Primer (downstream)TCCAACACTTCGAGAGGTCC  TaqMan Probe TGGTTAAGGTTGGCT-BHQ | 75 | 60 |

1. Dietert, K., et al., *mCLCA3 modulates IL-17 and CXCL-1 induction and leukocyte recruitment in murine Staphylococcus aureus pneumonia.* PLoS One, 2014. **9**(7): p. e102606.

2. Braun, J., et al., *Quantitative expression analyses of candidates for alternative anion conductance in cystic fibrosis mouse models.* J Cyst Fibros, 2010. **9**(5): p. 351-64.

3. Norris, M.D., et al., *Expression of N-myc and MRP genes and their relationship to N-myc gene dosage and tumor formation in a murine neuroblastoma model.* Med Pediatr Oncol, 2000. **35**(6): p. 585-9.

4. Giulietti, A., et al., *An overview of real-time quantitative PCR: applications to quantify cytokine gene expression.* Methods, 2001. **25**(4): p. 386-401.

5. García-Arévalo, M., et al., *Maternal Exposure to Bisphenol-A During Pregnancy Increases Pancreatic β-Cell Growth During Early Life in Male Mice Offspring.* Endocrinology, 2016. **157**(11): p. 4158-4171.
