# Supplementary figures and images for: The impact of disease and species differences on the intestinal CLCA4 gene expression
Source: J Mol Med (Berl). 2025 Apr 12;103(6):687–97. doi: 10.1007/s00109-025-02538-9 (PMC12141163; doi:10.1007/s00109-025-02538-9)

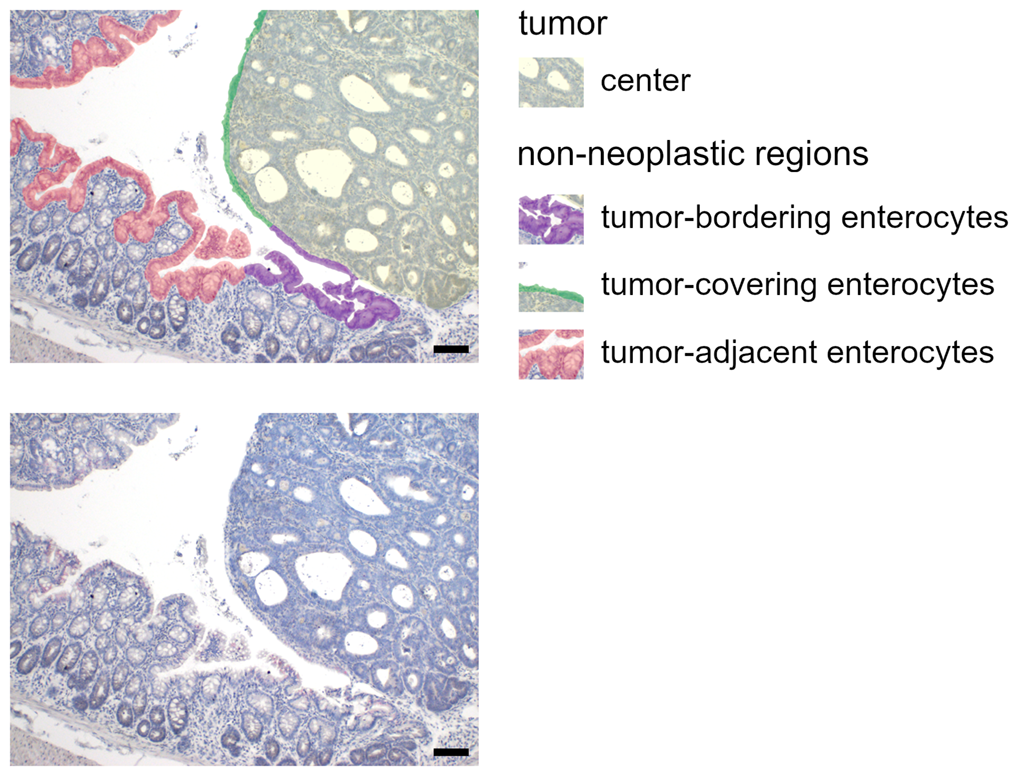

Supplement: Supplementary file 2 — S2. Figure – Depicted locations of cellular expression analysis of CLCA4 homologs in the CAC model. Yellow = tumor (center), green = non-neoplastic tumor-covering enterocytes, purple = non-neoplastic tumor-bordering enterocytes, red = non-neoplastic tumor-adjacent enterocytes. The same regions were analyzed in human CRC samples. Lower image = unannotated image, hematoxylin stained. Scale bars = 50 μm [file 109_2025_2538_Fig6_ESM.png]

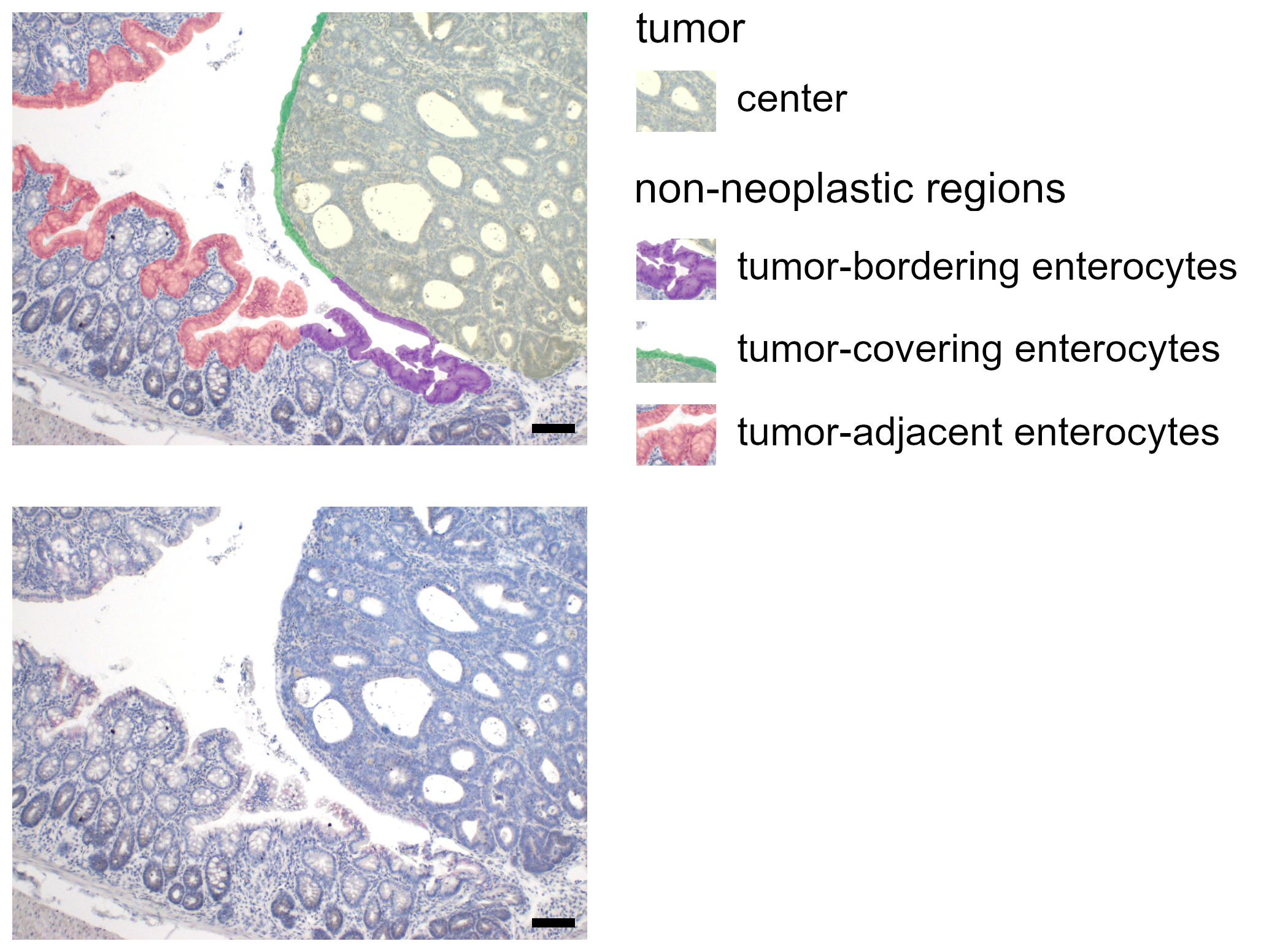

Supplement: Supplementary file 3 — High resolution image (TIF 9489 KB) [file 109_2025_2538_MOESM2_ESM.tif]

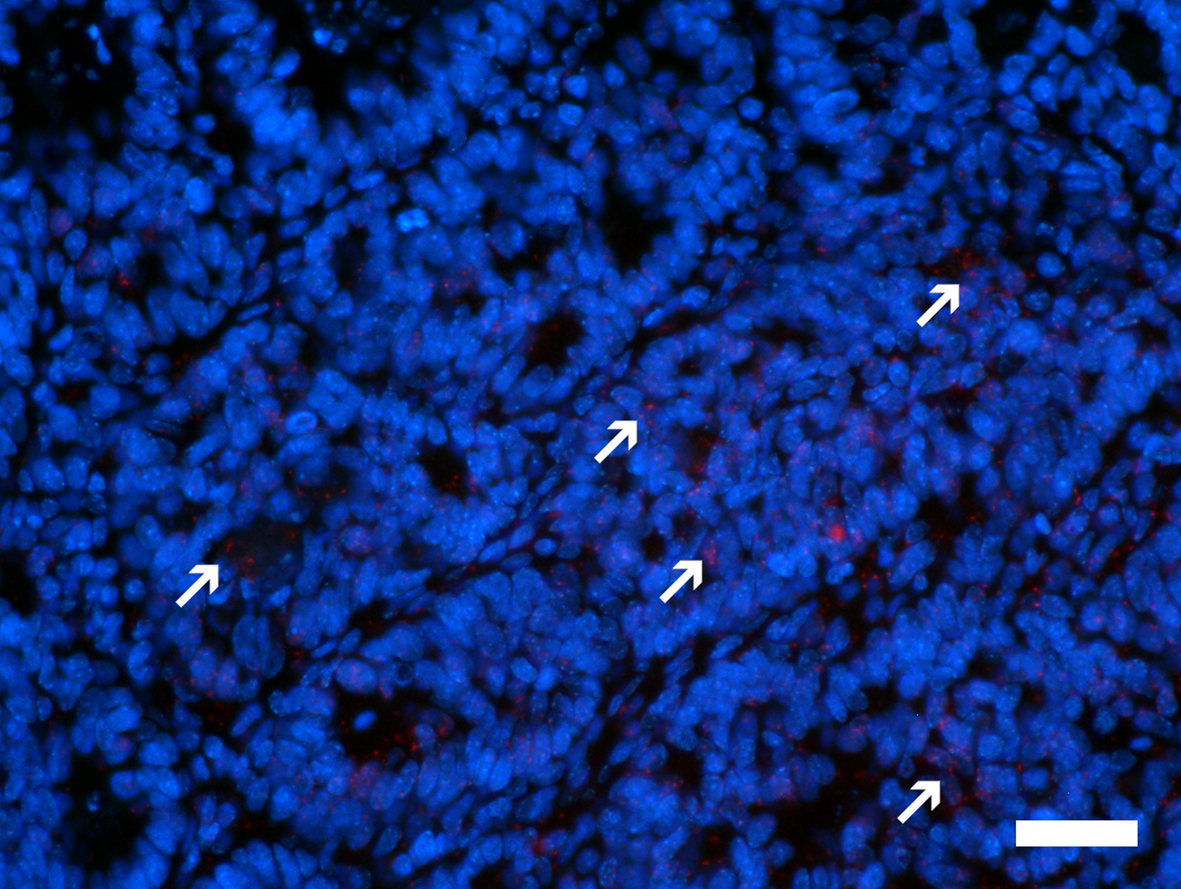

Supplement: Supplementary file 5 — S4.Figure – While no expression of Clca4b was detected in the depths of the neoplastic tissue in four mice with CAC via ISH, only a single mouseshowed moderate, diffuse Clca4b signals (red) in the tumor. Murine CAC model. Blue = Roti®-Mount Fluor-Care DAPI nucleus staining. Scale bar = 20 μm. [file 109_2025_2538_Fig7_ESM.png]

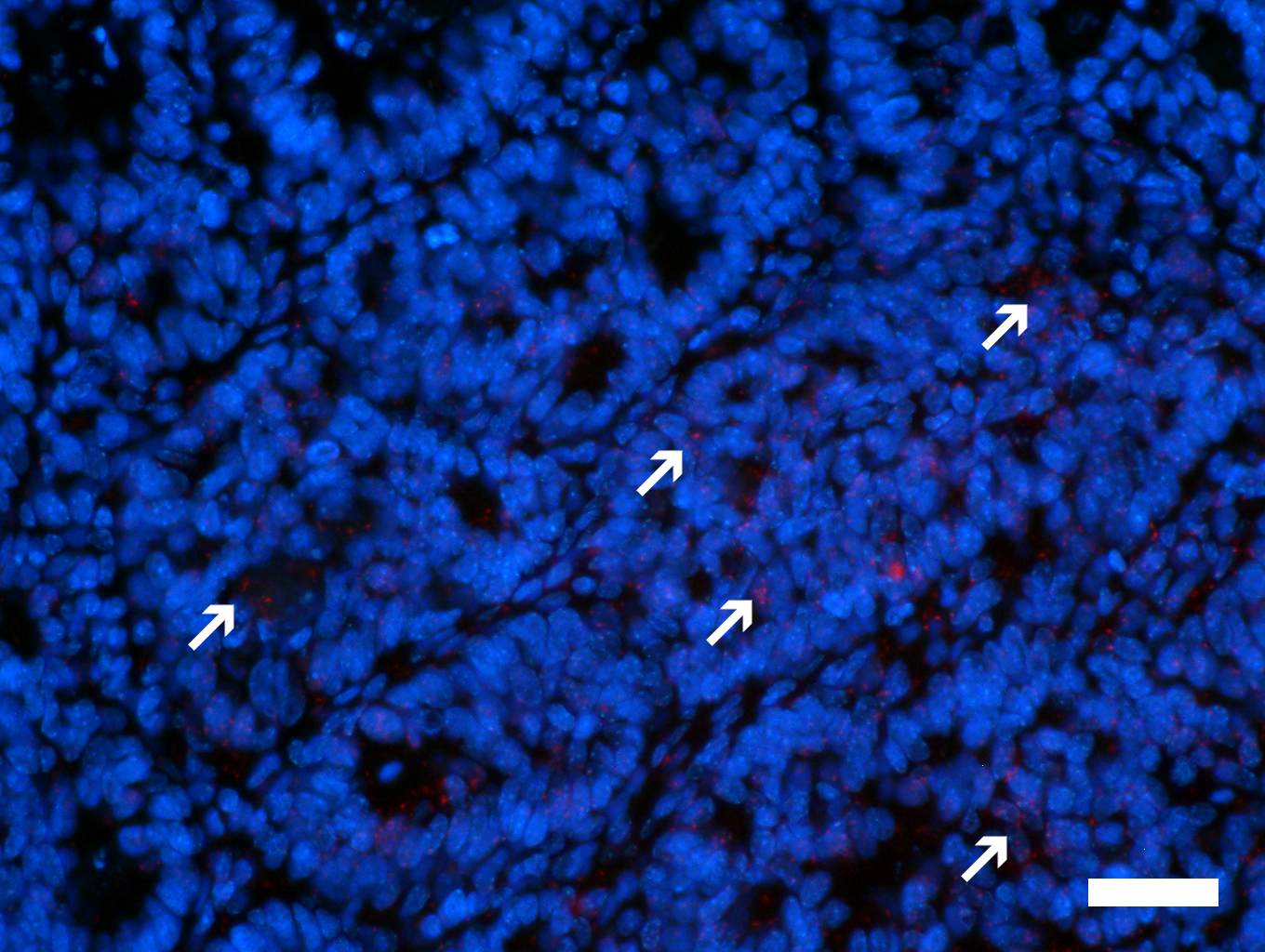

Supplement: Supplementary file 6 — High resolution image (TIF 4242 KB) [file 109_2025_2538_MOESM4_ESM.tif]

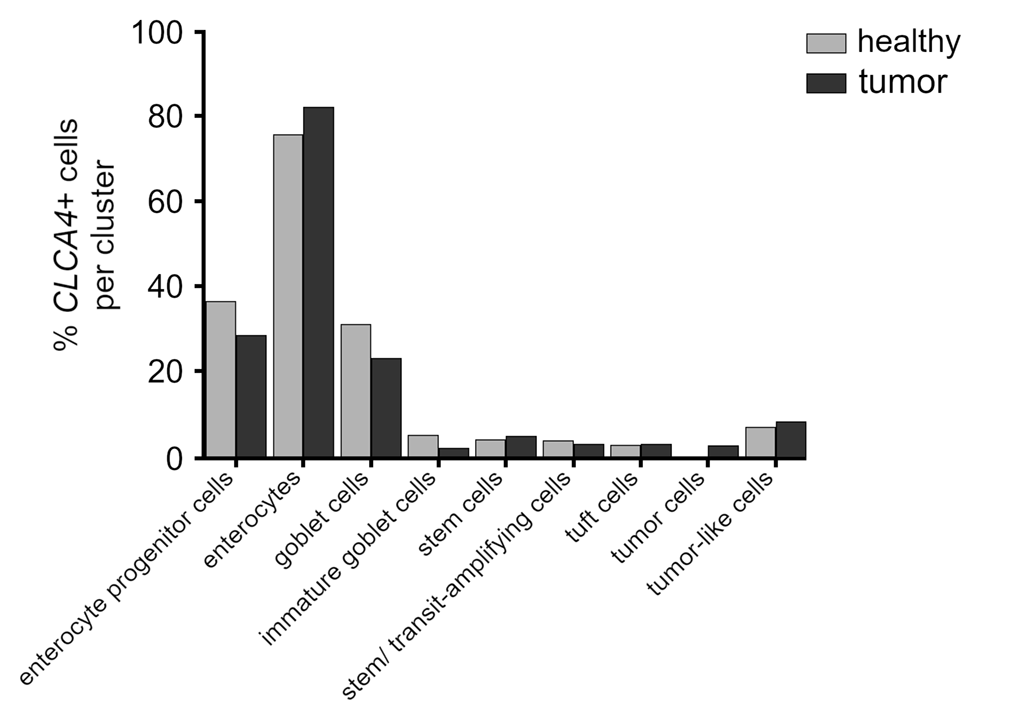

Supplement: Supplementary file 8 — S6. Figure - Percent of CLCA4 positive cells per cell population in human CRC compared to heathy adjacent tissue. Previously published [30] sc-RNA seq data were used for the calculation [file 109_2025_2538_Fig8_ESM.png]

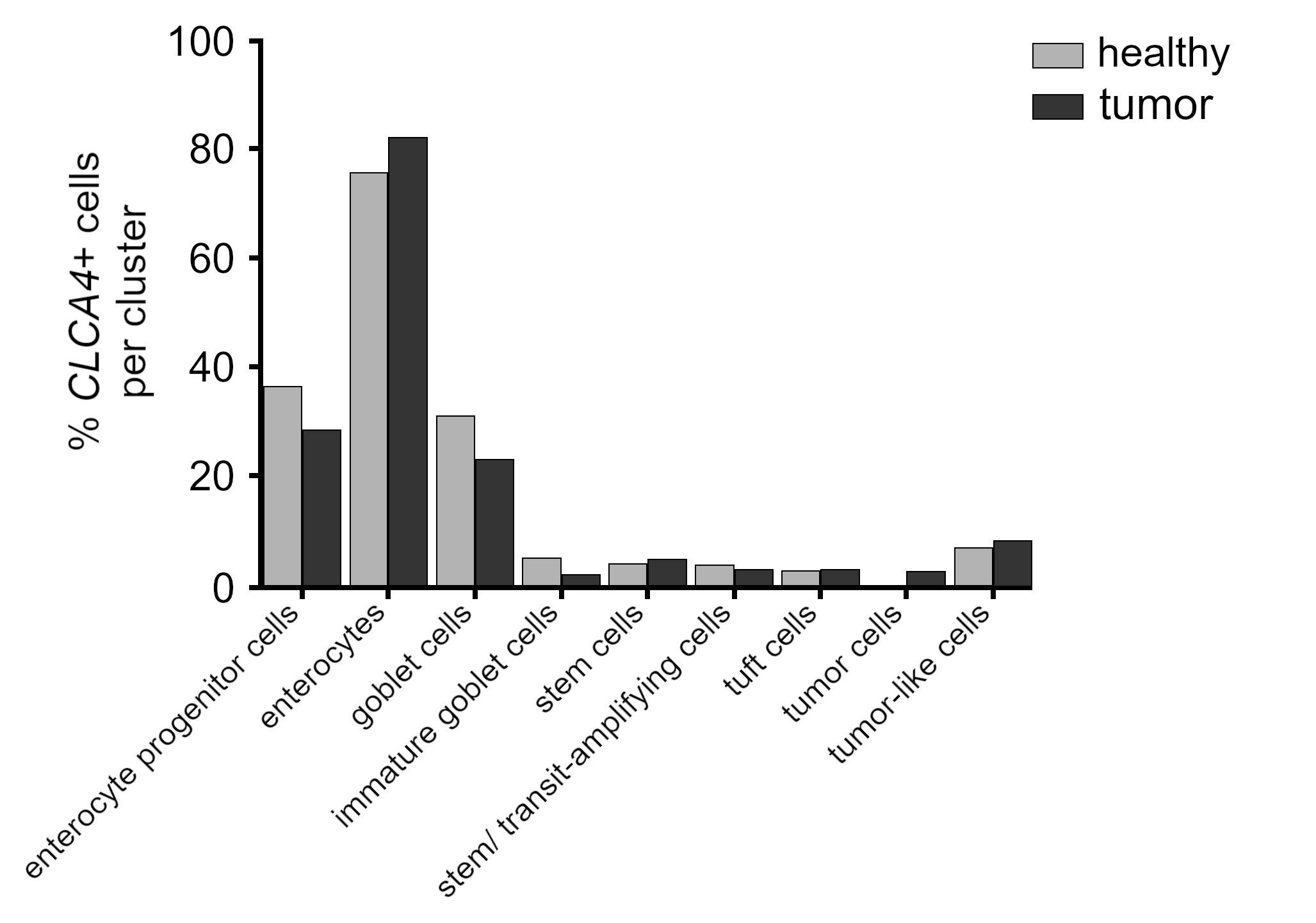

Supplement: Supplementary file 9 — High resolution image (TIF 8816 KB) [file 109_2025_2538_MOESM6_ESM.tif]
