# Supplementary material for: The impact of disease and species differences on the intestinal CLCA4 gene expression
Source: J Mol Med (Berl). 2025 Apr 12;103(6):687–97. doi: 10.1007/s00109-025-02538-9 (PMC12141163; doi:10.1007/s00109-025-02538-9)
Supplement: Supplementary file 4 — Supplementary file3 (DOCX 16 KB) [file 109_2025_2538_MOESM3_ESM.docx]

SUPPORTING INFORMATON

Table S3A

|  | tumor | non-neoplastic regions | | |
| --- | --- | --- | --- | --- |
|  | center | tumor-covering enterocytes | tumor-bordering enterocytes | tumor-adjacent enterocytes |
| animal 1 | - | ++ | ++ | + |
| animal 2 | - | +++ | not evaluable | ++ |
| animal 3 | - | ++ | not evaluable | + |
| animal 4 | - | +++ | +++ | ++ |
| animal 5 | - | ++ | not evaluable | ++ |

Table S3B

|  | tumor | non-neoplastic regions | | |
| --- | --- | --- | --- | --- |
|  | center | tumor-covering enterocytes | tumor-bordering enterocytes | tumor-adjacent enterocytes |
| animal 1 | - | + | +++ | +++ |
| animal 2 | - | + | not evaluable | +++ |
| animal 3 | - | + | not evaluable | ++ |
| animal 4 | - | +++ | +++ | ++ |
| animal 5 | + | ++ | not evaluable | +++ |
