# Supplementary material for: The impact of disease and species differences on the intestinal CLCA4 gene expression
Source: J Mol Med (Berl). 2025 Apr 12;103(6):687–97. doi: 10.1007/s00109-025-02538-9 (PMC12141163; doi:10.1007/s00109-025-02538-9)
Supplement: Supplementary file 7 — Supplementary file5 (DOCX 14 KB) [file 109_2025_2538_MOESM5_ESM.docx]

SUPPORTING INFORMATON

Table S5

|  | tumor | non-neoplastic regions | | |
| --- | --- | --- | --- | --- |
|  | center | tumor-covering enterocytes | tumor-covering enterocytes | tumor-covering enterocytes |
| patient 1 | - | +++ | +++ | +++ |
| patient 2 | + | ++ | +++ | +++ |
| patient 3 | + | not evaluable | ++ | ++ |
